# Perceived shifts in routine vaccine confidence during the COVID-19 pandemic in Kinshasa Province, DRC: A mixed-methods approach

**DOI:** 10.1371/journal.pgph.0004755

**Published:** 2025-07-16

**Authors:** Alix Boisson-Walsh, Patrick Ngimbi, Camille E. Morgan, Angela M. Stover, Nana Mbonze, Sarah Ntambua, Jolie Matondo, Marcel Yotebieng, Melchior M. Kashamuka, Linda James, Jonathan B. Parr, Samuel Mampunza, Peyton Thompson

**Affiliations:** 1 Division of Infectious Diseases, Department of Pediatrics, University of North Carolina, Chapel Hill, North Carolina, United States of America; 2 Faculté de Médecine, Université Protestante au Congo, Kinshasa, Democratic Republic of the Congo; 3 Department of Epidemiology, Gillings School of Global Public Health, The University of North Carolina, Chapel Hill, North Carolina, United States of America; 4 Department of Health Policy and Management, Gillings School of Global Public Health, Lineberger Comprehensive Cancer Center, The University of North Carolina, Chapel Hill, North Carolina, United States of America; 5 Faculté de Médecine, Université Protestante au Congo, Kinshasa, Democratic Republic of the Congo; 6 Faculté de Médecine, Université Protestante au Congo, Kinshasa, Democratic Republic of the Congo; 7 Faculté de Médecine, Université Protestante au Congo, Kinshasa, Democratic Republic of the Congo; 8 Division of General Internal Medicine, Department of Medicine, Albert Einstein College of Medicine, Bronx, New York, United States of America; 9 L‘École de Santé Publique, Université de Kinshasa, Kinshasa, Democratic Republic of the Congo; 10 Faculté de Médecine, Université Protestante au Congo, Kinshasa, Democratic Republic of the Congo; 11 Division of Infectious Diseases, UNC School of Medicine, University of North Carolina at Chapel Hill, Chapel Hill, North Carolina, United States of America; 12 Faculté de Médecine, Université Protestante au Congo, Kinshasa, Democratic Republic of the Congo; 13 Division of Infectious Diseases, Department of Pediatrics, University of North Carolina, Chapel Hill, North Carolina, United States of America; University of Michigan, UNITED STATES OF AMERICA

## Abstract

The COVID-19 pandemic negatively impacted routine immunizations worldwide, decreasing confidence in vaccination programs. We used mixed methods to examine changes in vaccine confidence from before to during the pandemic in HBV-negative adults in Kinshasa who were exposed to HBV in the household, and eligible for HBV vaccination. We measured changes in routine HBV vaccine confidence with a previously validated Shift in Vaccine Confidence (SVC) self-report measure that was verbally administered in Lingala (local language). We compared vaccination confidence before versus during the pandemic using Chi-square tests. We also interviewed participants and coded open-ended responses to the SVC scale to explore context-specific perceptions. From April 2022 to February 2023, we administered the SVC tool to a purposive sample of 41 participants: 7 vaccinees, 23 willing to receive HBV vaccine, and 11 refusers. Participants had a median age of 32 years, were predominantly affiliated with Revivalist churches, and most reported unemployment and no education beyond secondary school. We observed statistically significant declines across all five vaccine confidence domains when comparing responses before and during the pandemic (*p* < 0.01): vaccines prevent diseases (85.4%-68.3%), are safe (80.5%-46.3%), important for a child’s health (92.7%-87.8%) and one’s own health (87.8%-68.3%), and new vaccines carry no more risk than routine vaccines (78.1%-63.5%). Qualitative analysis identified four themes impacting uptake decisions: vaccine confidence, knowledge, risks, and external influences. Rising uncertainty about efficacy, safety, and distrust in the COVID-19 vaccine undermined vaccine confidence among our participants. Factors such as distrust in manufacturers and government, fear of side effects, perceived low illness risk, and inconvenient healthcare access contributed to low vaccine uptake. These insights underscore the pandemic’s impact on routine immunization and emphasize the need for consideration in future vaccination campaigns.

## 1. Introduction

The COVID-19 pandemic had a negative impact on public health domains worldwide, extending beyond the virus’s immediate consequences to broader, indirect effects on health systems, such as routine vaccination programs. At the height of the pandemic in 2020, the overall decline in coverage for all routine vaccines in low and middle-income countries ranged from 10-38%, compared to pre-pandemic coverage (2019). [1] Sub-Saharan Africa (SSA), in particular, experienced a significant decline in vaccination coverage. As of 2023, although global 3-dose hepatitis B vaccine (HBV) coverage is estimated to be 80%, coverage in SSA is estimated at 71% (and 67% in West and Central Africa), down from 75% in 2018. [2,3] This decline may be due to a combination of apprehension towards the presence of COVID-19 within healthcare settings and the emergence of associated vaccine hesitancy.

Over time, global awareness campaigns have successfully made routine vaccinations common practice in regions like SSA by focusing on building vaccine confidence. The pandemic caused worldwide debates about vaccine efficacy and safety, creating unprecedented turmoil driven by misinformation and stigmatization. [4] As we transition into a less severe phase of the COVID-19 pandemic, SSA countries continue to demonstrate lower vaccination uptake and relatively higher hesitancy than the global average. [5] A 2023 study reported that only 47% of respondents in the Democratic Republic of the Congo (DRC) would consider receiving a COVID-19 vaccine, marking the lowest acceptance rate among the eight African countries studied. [6] In addition, vaccination discussions have grown increasingly complex with the introduction of more vaccines into national immunization schedules. [7,8] This evolving landscape has significantly impacted the acceptance of COVID-19 vaccines and routine vaccinations, underscoring the urgent need for researchers and policymakers to assess and address the diminishing levels of trust and confidence in vaccines.

In 2019, the World Health Organization (WHO) recognized vaccine hesitancy as a critical global health concern. [9] The WHO defines vaccine hesitancy as the “delay or refusal to accept vaccines despite the availability of vaccination services” and characterizes the phenomenon through a conceptual framework known as the “3 Cs.” [8] This framework encompasses the dimensions of vaccine confidence, complacency, and convenience, providing valuable insights into the motivations of individuals situated at various points along the vaccine hesitancy spectrum. This spectrum ranges from individuals who readily accept vaccines with little doubt to those who adamantly reject them. The 3 Cs framework helps us comprehend the diverse range of individuals who fall between these two extremes and provides a tool for researchers and policymakers to address the multifaceted issue of vaccine hesitancy.

Applying the 3 Cs framework, [8] we used the previously validated Shift in Vaccine Confidence (SVC) tool [10] to measure changes in vaccine confidence among Kinshasa-based participants and to understand the factors that influenced vaccination confidence, uptake, and intention.

## 2. Materials and methods

### 2.1 Ethical Statement

All study participants provided written, informed consent to participate in the parent study and verbal consent to take part in this sub-study. Data were de-identified before uploading to REDCap, when sharing with the external translator and transcriber, and during analysis. We stored the data on a secure server with copies of the recorded transcripts. We received ethical approval from the Institutional Review Board at the University of North Carolina at Chapel Hill (IRB 21–1101) and the Université Protestante au Congo (IRB CEUPC 0091).

### 2.2 Study setting and survey tool

This study was an ancillary study to the “Horizontal and Vertical Transmission of HBV” (HOVER-HBV) study, which focused on investigating modes of HBV transmission within households in Kinshasa in February 2021. [11] Within the HOVER-HBV study, we observed low uptake of the HBV vaccine when offered free of charge to adults who were HBV-negative and exposed to HBV in their household. [11] In response to the decreased uptake, we developed and validated the content of the SVC tool [10], a standardized tool designed to assess changes in routine vaccine confidence resulting from the COVID-19 pandemic. We validated the content of the SVC tool by adhering to best practices for scale development [12–13] and refining it through multiple iterations. This process included cognitive interviews with content, technical, and context specialists, and pre-trial interviews in Kinshasa with study participants to ensure clarity, relevance, and usability. [10] The survey items measure five domains: vaccines prevent diseases, are safe, are important for a child’s health and one’s own health, and new vaccines carry no more risk than routine vaccines. Each domain is scored separately. The items included in the tool had a three-point rating scale (yes, don’t know, no) and open-ended questions. No changes were made to the wording of the items after the tool’s content validation. [10]

### 2.3 Design and population

In this cross-sectional mixed-methods study, we employed the SVC tool to survey a purposive sample of adults eligible for HBV vaccination within the parent study. [10] Eligible participants were HBV-negative, exposed to HBV in the household, and ≥18 years of age. DRC did not introduce the 3-dose infant HBV series to the national immunization schedule until 2009; [14] therefore, this adult population was not offered HBV vaccination until our study. We measured HBV vaccination status in three ways: vaccinee, willing to receive HBV vaccine, or refused. Individuals categorized as ‘willing to receive the HBV vaccine’ had scheduled an appointment for vaccination but did not attend their scheduled visit. Individuals who were ultimately vaccinated through the parent study were ‘vaccinees.’ Using a purposive sampling approach, we selected participants from each of the three groups in proportion to their actual response rates in the parent cohort. Our study staff approached participants by phone for this sub-study if they were offered an HBV vaccine in the parent study. If individuals consented to participate in the study, a research assistant would travel to the participant’s home on a pre-specified date and time to conduct an in-person interview.

### 2.4 Data collection

Two research assistants (JM, SN) with previous field interview experience and native to the region, received training on using the SVC tool. Interviews were conducted in Lingala (the local language) in a private space within the home and lasted between 45 and 60 minutes. We administered the interviews in two parts. The first was a structured survey format with three response options (yes, I don’t know, no). The second was a semi-structured interview format with open-ended questions. The interviewers received training to probe participants on determinants such as vaccine decision-making, uptake, and perception.

The study team used the REDCap (Research Electronic Data Capture) software to capture interview responses on a tablet. They were simultaneously audio recorded. A bilingual (French and Lingala) third-party service, ‘Centre Afro-Linguistique de Traduction, Interprétariat de Kinshasa,’ then translated and transcribed the audio recordings of the interviews to French for analysis.

### 2.5 Data analysis

#### 2.5.1 Quantitative analysis.

The SVC survey tool included five validated domains of vaccine confidence: vaccines prevent diseases, vaccines are important for a child’s health, vaccines are important for one’s own health, vaccines are safe, and new vaccines do not pose higher risk than existing vaccines. We interviewed participants at one timepoint during which they responded to interview questions in the framework of (1) before the pandemic and (2) during or currently. We then compared “before” and “during” pandemic responses to measure shifts in vaccine confidence using Pearson’s chi-square test. The responses were coded 0 for “No”, 1 for “I don’t know” and 2 for “Yes”. We reverse-coded one item’s response options (*do new vaccines pose more risk than old vaccines?*) so that higher scores indicated less confidence on all items. We used Stata/SE 16.1 to conduct all quantitative analyses.

#### 2.5.2 Qualitative analysis.

We uploaded the translated French transcripts into Dedoose V9.0.107, and three French-speaking team members (ABW, PN, CM) conducted thematic analysis using four interrelated steps: reading, coding and memo creation, data display, and data reduction. One author (ABW) read a sample of transcripts and developed a codebook based on responses by participants. Authors (ABW, PN, CM) then coded all transcripts and met iteratively to modify the codebook based on emergent codes, discuss major themes from the interviews, resolve codebook discrepancies, and establish intercoder reliability. We created a data display web using Visio to visualize the code’s links. We then used the “3 Cs” framework of vaccine hesitancy to conduct final data reduction within Dedoose and determine how the themes aligned with the framework. The final themes were established through consensus, ensuring their relevance to the research questions and analytical framework. We developed narrative summaries for each domain, which we present in the results section. These narrative summaries were shared routinely with the study team for feedback and modifications.

## 3. Results

### 3.1 Sample characteristics

**Fig 1:**
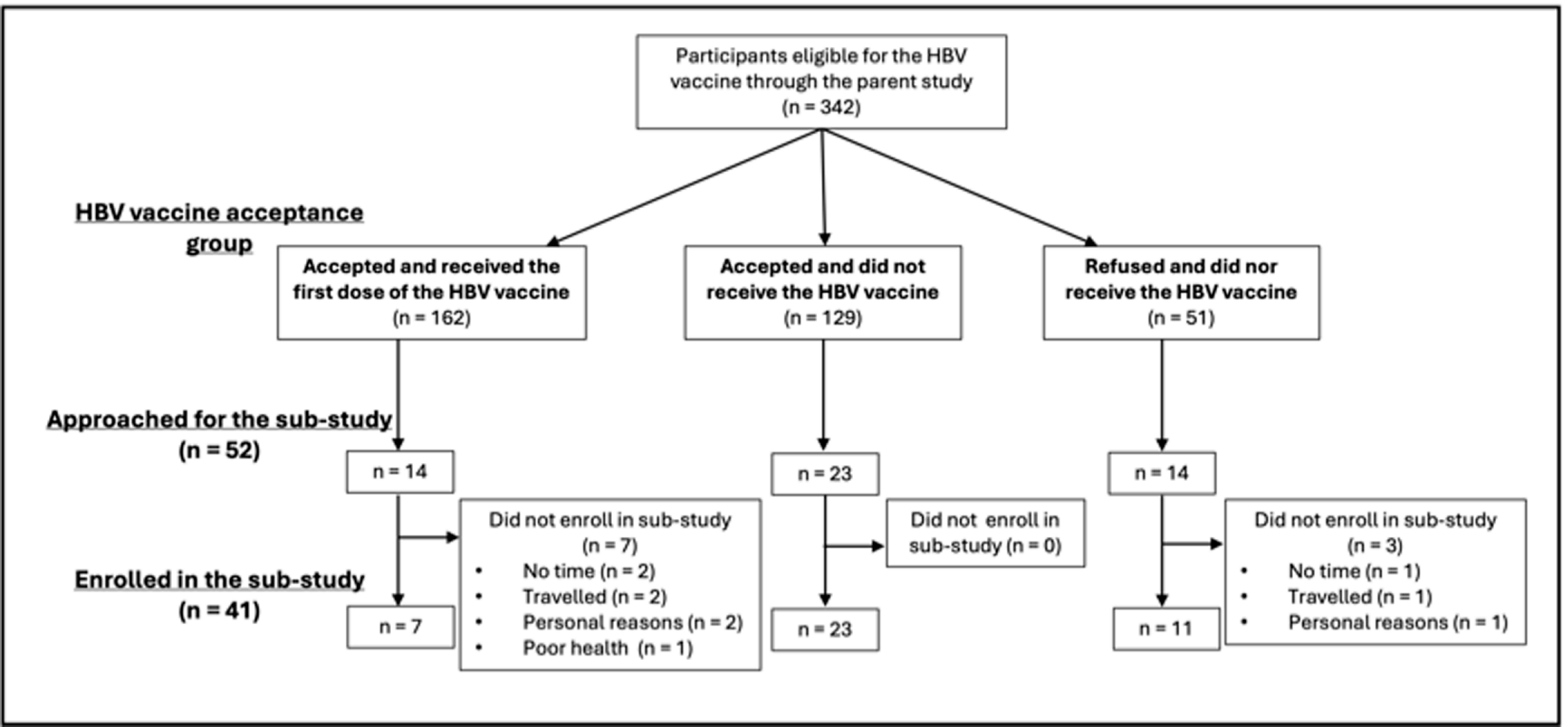
Recruitment and enrollment of parent and sub-study participants. We administered SVC to a purposive sample of 41 participants: 7 vaccinees, 23 vaccine acceptors who had not yet received the vaccine, and 11 vaccine refusers. The sample consisted of 22 men and 19 women, with a median age of 32 years (IQR: 22; 44) (Table 1). Most participants affiliated religiously with Revivalist churches (n = 29, 70.7%). The most common occupation response was unemployment (n = 18, 43.9%); nearly half of the participants finished secondary school with no university-level education (n = 17, 41.5%).

From April 1, 2022 to July 30, 2023, our study team approached 52 HOVER participants for enrollment in the sub-study: 14 vaccinees, 23 willing to receive HBV vaccine, and 14 who refused the vaccine. Among the vaccinees, seven participants refused to enroll – two did not have the time, two traveled during the study period, two for personal reasons, and one due to poor health. Among those who accepted but did not receive the vaccine, all 23 participants approached enrolled in the sub-study. Finally, among the 14 refusers approached for this study, one participant refused because they did not have the time, one traveled during the study period, and one refused for personal reasons. (Fig 1).

**Table 1:** Characteristics of Study Participants.

		M	(SD)
**Age**		32	(10.9)
		**N**	**(%)**
**Response category**			
	Accepted, did not receive	23	(56.1)
	Refusers	11	(26.8)
	Vaccinees	7	(17.1)
**Sex**			
	Male	22	(53.7)
	Female	19	(46.3)
**Religion**			
	Revivalist	29	(70.7)
	Catholic	7	(17.1)
	Other	3	(7.3)
	Protestant	1	(2.4)
	Kimbanguism	1	(2.4)
**Occupation**			
	No occupation	18	(43.9)
	Self-employed	9	(22.0)
	Salaried	8	(19.5)
	Other	4	(9.8)
	Student	2	(4.9)
**Education**			
	Finished secondary school	17	(41.5)
	3 years of university	9	(22.0)
	5 years of university	6	(14.6)
	Some secondary school	6	(14.6)
	No schooling or other	3	(7.3)

### 3.2 Quantitative results

**Fig 2:**
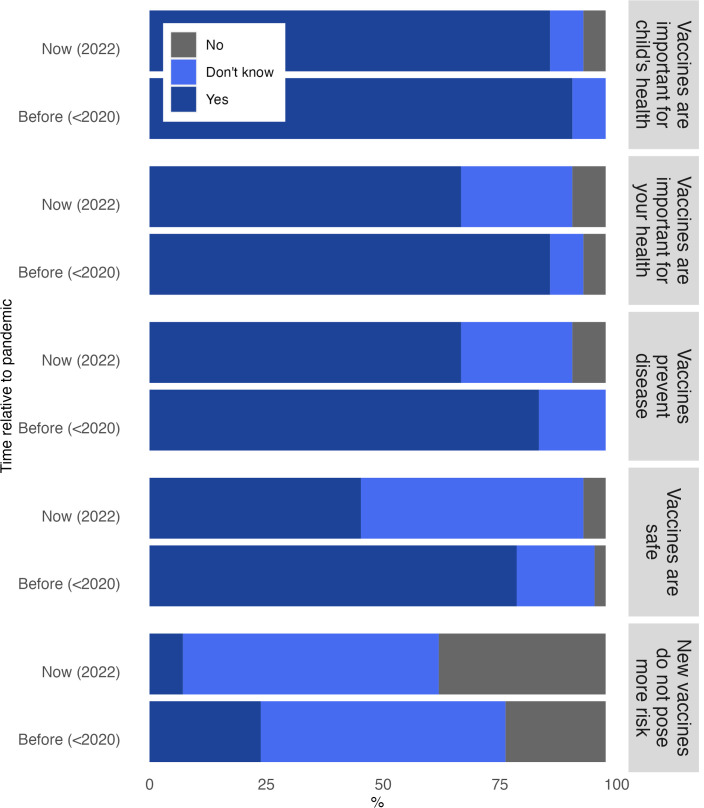
Shift in vaccine confidence from before to during the COVID-19 pandemic.

We observed a statistically significant shift from before the pandemic to during the pandemic in all five SVC items (Fig 2 and S1 Table). Before the pandemic, 85.4% of participants believed that vaccines effectively prevent diseases, compared to 68.3% during the pandemic (*p* < 0.001). Vaccines were perceived as more important for participants’ own health (87.8%) and children’s health (92.7%) before the pandemic than during the pandemic [own health [68.3%, (*p* < 0.01)], children’s health [87.8%, (*p* < 0.001)]. The most significant shift in perspective was that 80.5% of participants believed vaccines were safe before the pandemic, compared to 46.3% during the pandemic (*p* < 0.01). However, all but one of the respondents shifted their response to ‘I don’t know’ during the pandemic from ‘no’ before the pandemic. Finally, when asked whether new vaccines carry more risk than routine vaccines before the pandemic, 21.9% responded yes, compared to 36.6% who agreed with the statement during the pandemic (*p* < 0.001).

### 3.3 Qualitative results

During the interviews, participants discussed themes related to vaccine hesitancy and we organized the themes into sections utilizing the 3 Cs framework. We provide additional quotes in Table 2.

**Table 2:** Memorable quotes categorized by the three 3s of vaccine hesitancy.

**VACCINE CONFIDENCE**			
Shift in HBV vaccine confidence			
	*Health system distrust*	“Ma’am, I’m sorry, I’m afraid you’ll be giving me the covid vaccine instead of your HBV vaccine [if I attend my appointment at the health center].”	Female/ Willing to receive HBV vaccine
			
	*Government distrust*	“If only the government could earn back my trust. I have the impression that the government is more interested in money than in [its] people’s lives.”	Male/ Willing to receive HBV vaccine
		“I don’t trust our government’s policies.”	Male/ Willing to receive HBV vaccine
Low COVID-19 vaccine confidence			
	*Vaccine development*	“Yes, that’s what I just said about new vaccines, there’s too much trial and error, too many risks, especially with this covid vaccine story, there’s a lot to eat and drink here.”	Male/ Refused
			
		“I don’t trust the vaccine because it was developed too quickly, so I don’t trust the people or countries that made it. Another reason is that I’m afraid of the vaccine’s side effects when I get the shot. “	Female/ Willing to receive HBV vaccine
	*Secondary effects*	“Yes, we expect people to say that in the long run these vaccines will have negative effects on our health, so we’ll see in the long run if we can change any decisions.”	Male/ Refused
			
		“No, now we think [vaccines are] no longer important, because with this phase of the pandemic all vaccines are hacked.”	Female/ Willing to receive HBV vaccine
		“Yes, I will [receive the vaccine, let’s hope it doesn’t kill me]” *Female/ Received.*	Female/ Received HBV vaccine
	*Media portrayal*	“Today’s world has evolved so much that everything that happens in the world is tracked over time. The internet has taught us a lot about the famous covid vaccine, and how the world’s great powers are fighting to conquer the world with this vaccine: Americans, Europeans, Asians and even Africans are fighting for the covid vaccine.”	Male/ Refused HBV vaccine
**VACCINE COMPLACENCY**			
Risk of illness			
	*Natural defenses against diseases*	“I didn’t get the covid-19 vaccine, but still I’m alive and well.”	Male/ Willing to receive HBV vaccine
			
	*Vaccines received during childhood protect against diseases*	“My child will already be protected with the vaccines he will receive during his CPS [Childhood Vaccine Schedule].”	Male/ Willing to receive HBV vaccine
			
	*Recognition of elevated disease vulnerability*	“Like all diseases, it is best to vaccinate to prevent the disease. I don’t have any clear risks, but I’m strongly convinced that there are risks in not getting vaccinated. Among other things, antibodies will weaken in the absence of a vaccine.”	Male/ Willing to receive HBV vaccine
	*Enhanced vaccine acceptance for children*	“Personally, I don’t trust this covid vaccine in children, especially as its side effects are not well known.”	Male/Refused HBV vaccine
Shift in decision-making			
	*Religious leaders decision*	“.. the vaccine is against our religious beliefs; my religion does not allow us to benefit from vaccinations.”	Female/ Refused HBV vaccine
	*Partner’s responsibility for CPS decisions*	“I don’t know, since it’s the mom who brings the kids to CPS, I’m not too interested.”	Male/ Refused HBV vaccine
	*No shift – autonomous decision-making*	“No, the decision to vaccinate comes from me [alone].”	Male/ Received HBV vaccine
**VACCINE CONVENIENCE**			
Accessibility challenges			
	*Distance and affordability*	“The facility that provides the vaccines is too far from my home, and to get there I have to take 3 cabs to go there and 3 more to return. Right from the start I told you that I would never get there…”	Female/ Refused HBV vaccine
	*Time*	“I don’t have time to get to the health center [to be vaccinated], I’m a finalist at school and we do 2 shifts morning and evening at school, so it’s hard for me to get to the health center for the vaccination. Maybe I can take advantage of the time I have during vacations to get vaccinated.”	Male/ Willing to receive HBV vaccine
		“Ma’am Nurse, you see that you came to my house on Sunday, simply because of my work schedule. I work from Monday to Saturday.”	Female/ Willing to receive HPV vaccine
	*CPS = Calendrier des Vaccinations Pour la Petite Enfance (Childhood Vaccine Schedule)		

#### 3.3.1 Vaccine confidence.

A prominent recurring theme that emerged during the interviews centered around vaccine confidence, encompassing both established HBV vaccines and new COVID-19 vaccines. Both the structured survey and semi-structured interview segments revealed that a decline in HBV vaccine confidence was linked to the COVID-19 pandemic and associated vaccine hesitancy.

A noteworthy finding was that several participants reported postponing their HBV vaccines out of fear. They expressed concerns that the staff would administer the COVID-19 vaccine instead of the HBV vaccine.

“Ma’am, I’m sorry, I’m afraid you’ll be giving me the COVID vaccine instead of your HBV vaccine [if I attend my appointment at the health center].” *Female/ Willing to receive HBV vaccine*

A few participants also expressed skepticism about the government’s vaccine policies.

“If only the government could earn back my trust. I have the impression that the government is more interested in money than in [its] people’s lives.” *Male/ Willing to receive HBV vaccine*

Several factors contributed to vaccine hesitancy regarding the COVID-19 vaccine, with participants highlighting various reasons for distrusting the vaccine. These included concerns about the rapid development process, mistrust in vaccine manufacturers, fear of potential side effects leading to perceived severe outcomes, doubts about the vaccine’s efficacy, and the influence of media portrayal.

“I don’t trust the vaccine because it was developed too quickly, so I don’t trust the people or countries that made it. Another reason is that I’m afraid of the vaccine’s side effects when I get the shot.” *Female/ Willing to receive HBV vaccine*

Notably, even among participants who expressed willingness to receive a future COVID-19 vaccine, an overt skepticism toward the vaccine remained.

“Yes, I will [receive the COVID-19 vaccine], let’s hope it doesn’t kill me.” *Female/ Received HBV vaccine*

#### 3.3.2 Vaccine complacency.

Several comments related to complacency revealed varying levels of knowledge about the risks of not getting vaccinated and a tendency to delegate vaccine decision-making to others. The quantitative findings indicated that participants generally acknowledged the risk of illness if they or their children remained unvaccinated. However, further exploration during the open-response portion showed that many participants perceived a relatively low risk of contracting both infections (HBV and COVID-19). Some adopted a mid-risk belief, waiting until the pandemic ends before seeking vaccination, while a few perceived the risk of illness as high.

Participants provided several reasons for perceiving a low risk, with some believing that the diseases may not exist. Some felt confident in their protection from new diseases due to vaccinations received during childhood, while others simply held the belief that they were already healthy as they were. One participant noted:

“I didn’t get the COVID-19 vaccine, but still I’m alive and well.” *Male/ Willing to receive HBV vaccine*

An emergent theme during interviews was the delegation of vaccine decision-making to others, including religious leaders or a partner. One participant cited:

“.. the vaccine is against our religious beliefs; my religion does not allow us to benefit from vaccinations.” *Female/ Refused*

On the other hand, it is worth noting that some participants expressed strong personal ownership of vaccine decisions, firmly stating that they alone can make such choices.

“No, the decision to vaccinate comes from me [alone].” *Male/ Received HBV vaccine*

We found that when it came to children, participants were much more steadfast in their beliefs, whether regarding the assumption that routine vaccines were necessary for protecting infants or their deeper concerns about the COVID-19 vaccine’s potential risk of harm to their infants.

#### 3.3.3 Vaccine convenience.

Many participants were willing to receive the HBV vaccine but faced challenges attending a clinic visit, even when the vaccine was offered free and the clinics had walk-in appointments. Obstacles included lack of time, long distances to the clinic, and concerns about transportation costs. One participant expressed their frustrations:

“The facility that provides the vaccines is too far from my home, and to get there I have to take 3 cabs to go there and 3 more to return. Right from the start I told you I would never get there…” *Female/ Refused*

While many participants who didn’t receive a vaccine cited convenience challenges, five of the seven who received the vaccine reported no difficulties in accessing and receiving it.

## 4. Discussion

This study used the SVC tool [10] to understand changes in vaccine confidence stemming from the pandemic and to explore vaccine hesitancy dynamics among Kinshasa-based participants. After observing a clear shift in vaccine confidence among our sample, we investigated the underlying motivations behind vaccine hesitancy.

SVC tool-led interviews identified various themes within the vaccine confidence domain, encompassing both the well-established HBV vaccine and the novel COVID-19 vaccine. The dynamic relationship between diminishing confidence in HBV vaccination, the influence of the COVID-19 pandemic, and the emergence of vaccine hesitancy was apparent across our structured and semi-structured interview sessions. A notable discovery surfaced as several participants disclosed their decision to postpone HBV vaccinations due to concerns of inadvertently receiving the COVID-19 vaccine instead. The intense focus on COVID-19 vaccines and the associated uncertainties regarding their safety and efficacy may have fueled these fears. Our quantitative findings further substantiated this sentiment, revealing that participants felt more uncertain about vaccine safety during the pandemic than before. Clear and transparent communication strategies are critical to address these fears. Drawing from a 2022 Ghana-based study investigating factors influencing vaccine acceptance among 50,000 participants, health workers emerged as the most trusted source of information about COVID-19 and its corresponding vaccine. [15] Healthcare providers therefore play a pivotal role in reestablishing and reinforcing vaccine confidence. [15,16] Healthcare providers can proactively educate patients about the distinct vaccination processes, emphasizing the separation of COVID-19 and HBV or other routine vaccination procedures. Simultaneously, health facilities may prioritize efforts to enhance the uptake of COVID-19 vaccines and integrate them into the routine immunization schedule, aligning with the latest WHO COVID-19 vaccine uptake strategy. [16]

In addition to concerns related to vaccine confidence, our study also revealed elements of vaccine complacency. Some participants perceived a low risk of contracting infectious diseases, including HBV and COVID-19, which led them to postpone or forego vaccination. This complacency highlights the importance of continuous public health education and awareness campaigns. [17] Efforts should focus on conveying public health messaging about the importance of vaccination and provide clear and accessible information about the diseases, their transmission, their severity, and potential consequences, especially during and despite the pandemic. [17–19] By maintaining consistent public health education and awareness efforts, we can empower individuals to make informed decisions about their health and the health of their communities.

Another significant finding was the delegation of vaccine decision-making to religious leaders or partners. Over the past few years of the pandemic, a persistent impediment to COVID-19 vaccination has been identified in the form of resistance from religious leaders. [20–22] In the DRC, religious leaders consistently discouraged COVID-19 vaccination, advocating instead for reliance on faith and prayer as sufficient protection against the virus. [23] However, as time progressed, centralized leadership within more traditional religious structures began actively promoting the COVID-19 vaccine, particularly following political leadership endorsements. [22] In a 15-country study across Sub-Saharan Africa, researchers found significantly lower full immunization coverage among Muslims compared to Christians in nine of the countries. The study highlighted the crucial impact of leadership buy-in, emphasizing the distinct levels of support between the two religious groups. The research emphasized the influential role of religious leaders in promoting immunization efforts. [24] Evidence suggests that strategies involving community leaders are effective in mitigating the spread of misinformation and infectious diseases. [25–27] In our study sample, 70% of participants were associated with the Revivalist Church, comprising independent entities without centralized leadership. This finding aligns with findings from the parent study and may indicate a broader trend in Kinshasa, where the prominence of this form of religion is increasing. [11] While more traditional religious entities hold more significant control over messaging from a central body, the Revivalist Church’s decentralized nature presents challenges in disseminating consistent vaccine-related messages. Consequently, advocates and public health authorities must develop innovative grassroots-level approaches to engage with these emerging religious entities and address vaccine-related misconceptions at the community level.

The lack of vaccine convenience emerged as a barrier to HBV vaccination, with participants citing distance to vaccination facilities and time constraints as reasons for not receiving vaccines. Among the seven individuals who received the HBV vaccine, 71.4% (n = 5) reported no difficulty accessing facilities. This suggests the possibility of a mental barrier to convenience rather than a logistical one. Strategies to address this barrier and boost vaccine uptake include offering flexible hours and providing transportation options for those facing geographical or financial constraints. [27–30] Community-based outreach and education programs involving religious leaders can also help raise awareness and address logistical challenges.

### 4.1 Limitations

Despite efforts to emphasize participant engagement during interview training, interviewers received a relatively high frequency (12% within the vaccine confidence domain) of ‘Do not know’ responses recorded for the survey questions, suggesting a pattern of disinterest, distraction, or some other factors among respondents. Understanding the impact of the pandemic on routine vaccines remains crucial for enhancing global vaccine distribution systems.

Furthermore, we drew our study sample from a cohort of participants already enrolled in a parent HBV study. As a result, we could not employ a random sampling approach and instead relied on a purposive sample. Consequently, it is essential to acknowledge that the results obtained in this study may not apply to the broader population of Kinshasa Province. Our smaller sample size, drawn from the participant cohort, may have limited statistical power and generalizability. Future research with a larger sample size and a randomized sampling approach is warranted to strengthen the findings. Due to the small sample size, our study utilizes bivariate comparisons and therefore does not control for potential confounders such as exposure to misinformation, healthcare access, and prior vaccine experiences. Additionally, our sample was atypical in that participants were offered the HBV vaccine free of charge. While this unique aspect of our study might suggest that these participants have a higher level of HBV knowledge or acceptance, it is important to emphasize that our primary focus was evaluating shifts in perceived confidence regarding routine vaccines. In this context, the baseline knowledge of HBV was less critical. Additionally, our study was susceptible to recall bias when evaluating participants’ perceptions of the period before the pandemic. This susceptibility arose from the extended duration of the pandemic and the significant time that had transpired since the pre-pandemic period. We recognized the potential for this bias during our data analysis and considered it when interpreting the findings. Finally, the use of three languages (English, French, and Lingala) during data collection and analysis may have led to a loss of some language or cultural nuances.

## 5. Conclusions

Leveraging the SVC tool in a mixed methods approach, we assessed shifts in routine HBV vaccine confidence during the pandemic. We found increasing uncertainty about the efficacy of vaccines in preventing disease, concerns about the safety of new vaccines, and low confidence in these vaccines among participants. We also found decreased confidence in routine vaccines due to the COVID-19 pandemic and several factors contributing to distrust of the COVID-19 vaccine, including distrust of manufacturers and the government and fear of severe side effects. In addition, the perceived low risk of illness and the lack of convenience in accessing healthcare services contributed to low vaccine uptake among our study participants. These findings inform the pandemic’s influence on routine immunization and require due consideration in future vaccination campaigns.

## Supporting information

S1 Table Vaccine confidence shifts before vs during the pandemic. (DOCX)

S1 Checklist Inclusivity in global research. (DOCX)
