# Correction: Perceived shifts in routine vaccine confidence during the COVID-19 pandemic in Kinshasa Province, DRC: A mixed-methods approach

**DOI:** 10.1371/journal.pgph.0006782

**Published:** 2026-06-29

**Authors:** Alix Boisson-Walsh, Patrick Ngimbi, Camille E. Morgan, Angela M. Stover, Nana Mbonze, Sarah Ntambua, Jolie Matondo, Marcel Yotebieng, Melchior M. Kashamuka, Linda James, Jonathan B. Parr, Samuel Mampunza, Peyton Thompson

There are errors in the Funding statement. The correct Funding statement is as follows: Peyton Thompson reports financial support from the American Society of Tropical Medicine and Hygiene (Postdoctoral Fellowship in Tropical Infectious Diseases – no grant number), and grant funding from Novavax Inc (2019nCoV-301) and the National Institutes of Health (K08AI148607). Dr. Thompson also reports non-financial support from Abbott Diagnostics (no grant number). Camille Morgan reports grant funding from the National Institutes of Health (F30AI169752 and D43TW009340), and support from the UNC Graduate School Royster Fellowship (no grant number) and the Infectious Disease Society of America’s Grants for Emerging Researchers/Clinicians Mentorship Program (no grant number). Angela Stover reports unrelated grant funding from non-profit and for-profit organizations (UroGen Pharma Ltd and Pfizer). She also reports speaking and lecture fees from Pfizer and UroGen Pharma Ltd. Jonathan B. Parr reports grant funding from Gilead Sciences Inc (IN-US-320-5626), non-financial support from Abbott Diagnostics, and consulting or advisory roles with Zymeron Corp. Marcel Yotebieng reports grant funding from the National Institutes of Health (R01HD087993, U54CA254568, U01AI096299, R01HD105526) and non-financial support from Abbott Diagnostics. Alix Boisson-Walsh reports consulting or advisory work with the World Health Organization.
