# Supplementary material for: Perceived shifts in routine vaccine confidence during the COVID-19 pandemic in Kinshasa Province, DRC: A mixed-methods approach
Source: PLOS Glob Public Health. 2025 Jul 16;5(7):e0004755. doi: 10.1371/journal.pgph.0004755 (PMC12266390; doi:10.1371/journal.pgph.0004755)
Supplement: S1 Table — (DOCX) [file pgph.0004755.s001.docx]

| **S1 Table.** Vaccine confidence shifts before vs during the pandemic | | | | | |  |  |
| --- | --- | --- | --- | --- | --- | --- | --- |
| Vaccines prevent disease | | |  |  |  |  |  |
|  |  |  | DURING THE PANDEMIC | | |  |  |
|  |  |  | **No** | **Don't know** | **Yes** |  |  |
|  | BEFORE THE PANDEMIC | **No** | 0 | 0 | 0 | 0 |  |
|  |  | **Don't know** | 0 | 6 | 0 | 6 |  |
|  |  | **Yes** | 3 | 4 | 28 | 35 |  |
|  |  |  | 3 | 10 | 28 | 41 |  |
|  |  |  | Pearson chi2 = | 21.79 | **Pr =** | **0.000***** |  |
| Vaccines are important for child's health | | | |  |  |  |  |
|  |  |  | **No** | **Don't know** | **Yes** |  |  |
|  | BEFORE THE PANDEMIC | **No** | 0 | 0 | 0 | 0 |  |
|  |  | **Don't know** | 0 | 2 | 1 | 3 |  |
|  |  | **Yes** | 2 | 1 | 35 | 38 |  |
|  |  |  | 2 | 3 | 36 | 41 |  |
|  |  |  | Pearson chi2 = | 16.83 | **Pr =** | **0.000***** |  |
| Vaccines are important for your health | | | |  |  |  |  |
|  |  |  | **No** | **Don't know** | **Yes** |  |  |
|  | BEFORE THE PANDEMIC | **No** | 1 | 1 | 0 | 2 |  |
|  |  | **Don't know** | 0 | 2 | 1 | 3 |  |
|  |  | **Yes** | 2 | 7 | 27 | 36 |  |
|  |  |  | 3 | 10 | 28 | 41 |  |
|  |  |  | Pearson chi2 = | 10.59 | **Pr =** | **0.032**** |  |
| New vaccines do not pose more risk | | | |  |  |  |  |
|  |  |  | **No** | **Don't know** | **Yes** |  |  |
|  | BEFORE THE PANDEMIC | **No** | 7 | 1 | 1 | 9 |  |
|  |  | **Don't know** | 3 | 19 | 0 | 22 |  |
|  |  | **Yes** | 5 | 3 | 2 | 10 |  |
|  |  |  | 15 | 23 | 3 | 41 |  |
|  |  |  | Pearson chi2 = | 19.87 | **Pr =** | **0.001***** |  |
| Vaccines are safe | | |  |  |  |  |  |
|  |  |  | **No** | **Don't know** | **Yes** |  |  |
|  | BEFORE THE PANDEMIC | **No** | 0 | 0 | 1 | 1 |  |
|  |  | **Don't know** | 0 | 7 | 0 | 7 |  |
|  |  | **Yes** | 2 | 13 | 18 | 33 |  |
|  |  |  | 2 | 20 | 19 | 41 |  |
|  |  |  | Pearson chi2 = | 9.68 | **Pr =** | **0.046**** |  |
|  |  |  |  |  |  |  |  |
